# Integrated Plastic Microfluidic Device for Heavy Metal Ion Detection

**DOI:** 10.3390/mi14081595

**Published:** 2023-08-13

**Authors:** Myrto Kyriaki Filippidou, Aris Ioannis Kanaris, Evangelos Aslanidis, Annita Rapesi, Dimitra Tsounidi, Sotirios Ntouskas, Evangelos Skotadis, George Tsekenis, Dimitris Tsoukalas, Angeliki Tserepi, Stavros Chatzandroulis

**Affiliations:** 1 Institute of Nanoscience and Nanotechnology, NCSR ‘‘Demokritos’’, 15341 Aghia Paraskevi, Greece; m.filippidou@inn.demokritos.gr (M.K.F.); a.tserepi@inn.demokritos.gr (A.T.); 2 Department of Applied Sciences, National Technical University of Athens, 15780 Zografou, Greece; aslanidisvag@gmail.com (E.A.); evskotad@central.ntua.gr (E.S.); dtsouk@central.ntua.gr (D.T.); 3 Biomedical Research Foundation of the Academy of Athens, 11527 Athens, Greecegtsekenis@bioacademy.gr (G.T.)

**Keywords:** Lab on a Chip, microfluidics, microfabrication, Kapton, heavy metal ion detection, DNAzyme, nanoparticles, biosensor

## Abstract

The presence of heavy metal ions in soil, air and water constitutes an important global environmental threat, as these ions accumulate throughout the food chain, contributing to the rise of chronic diseases, including, amongst others, cancer and kidney failure. To date, many efforts have been made for their detection, but there is still a need for the development of sensitive, low-cost, and portable devices able to conduct on-site detection of heavy metal ions. In this work, we combine microfluidic technology and electrochemical sensing in a plastic chip for the selective detection of heavy metal ions utilizing DNAzymes immobilized in between platinum nanoparticles (PtNPs), demonstrating a reliable portable solution for water pollution monitoring. For the realization of the microfluidic-based heavy metal ion detection device, a fast and easy-to-implement fabrication method based on the photolithography of dry photosensitive layers is proposed. As a proof of concept, we demonstrate the detection of Pb^2+^ ions using the prototype microfluidic device.

## 1. Introduction

Heavy metal ions, such as lead (Pb^2+^), cadmium (Cd^2+^), and mercury (Hg^2+^), constitute one class of environmental pollutants whose high chemical stability and low biodegradability results in their bioaccumulation, which negatively impacts human health and can lead to cancer, kidney failure, diseases of the immune system, and neurodegenerative diseases, as well as poses a threat to the environment [1,2,3,4]. Water testing for the presence of heavy metals is thus of paramount importance in producing drinking water of high quality that meets the internationally accepted limits. The methods currently employed for the detection of heavy metal ions include spectroscopic techniques (e.g., atomic absorption spectroscopy (AAS) and inductively coupled plasma mass spectroscopy (ICP-MS)), as well as other analytical methods such as high-performance liquid chromatography (HPLC). These methods are characterized by high accuracy and sensitivity, but they require bulky and expensive equipment, available only in analytical laboratories, while sample handling is time-consuming and necessitates highly trained personnel. Most importantly, these techniques do not cater to continuous sample monitoring [5,6].

Biosensing approaches and their fabrication methods [7] fulfill the criteria for rapid, inexpensive analysis of human [8], water [9], and food samples [10], while their integration into complete Lab on a Chip (LOC) systems [11,12,13,14] allows their implementation at the point of interest [5]. Such approaches require the use of microfluidic technology for sample introduction, pretreatment, and analysis and have brought a revolution in the fabrication of devices that are compact enough to be employed in field detection applications [15]. LOCs can be classified into three categories, depending on the methodology employed to drive the sample through the platform: paper-based microfluidics, digital microfluidics (DMF), and continuous-flow microfluidics [16]. Paper-based devices are based on the capillary action induced by paper, which can enable passive liquid motion, and their fabrication methods are also easy and cost-effective [6,17,18,19]. The signal readout in such devices is usually colorimetric, lacking the sensitivity and the dynamic range resolution required to meet the needs of numerous applications in food safety and environmental monitoring [17]. Moreover, it is difficult to control the liquid flow, which severely compromises the accuracy and reliability of such devices. Furthermore, the integration of several consecutive processes necessary in real sample analysis (i.e., sample pretreatment and mixing with buffer) is challenging. By contrast, the performance of digital microfluidic LOCs is far superior to that of paper-based devices, which can be attributed in part to the high flexibility in droplet processing, but most importantly to the increased sensitivity attained by DMF LOCs due to the employment of electrochemical detection schemes [20]. On the downside, multiple materials and fabrication steps are required for the fabrication of DMF LOCs, a considerable and difficult-to-overcome obstacle for their up-scaled production at a low cost [21]. Lastly, continuous flow, microfluidic-based LOCs present a number of significant advantages over the other two approaches, and several examples of such devices for heavy metal ion detection have been proposed in recent review paper reports [5]. In particular, LOC devices can be (a) fully integrated, (b) coupled with several detection methods, (c) customized according to the application envisioned, and (d) incorporate novel pumping methods such as photothermal pumping to enable flow control [22]. Nevertheless, and despite the significant advancements made, the design and the development of easy-to-fabricate, highly integrated devices which enable multiplexed heavy metal ion detection with high sensitivity and specificity remains challenging.

Electrochemical detection schemes based on nanoparticle (NP)-modified sensors, coupled with the use of DNAzymes as biorecognition molecules, are a promising solution to the aforementioned challenges. The use of NPs as a transducer layer enhances surface-volume ratios attained and sensor conductivity and permits the label-free detection of analytes. Moreover, nanoparticle-based sensors can be easily fabricated using sputtering techniques [23]. Nanoparticles, if coupled with DNAzymes, significantly improve the sensor’s sensitivity and specificity [1] due to their ability to catalyze their respective reactions with multiple turnovers and the selective formation of coordinated complexes with metal ions. Moreover, DNAzymes possess several beneficial properties, including good chemical stability, ease of modification with signaling molecules, and synthetic accessibility. DNAzyme-based sensors have been reported for the detection of many metal ions in complex matrices, such as Pb^2+^, UO_2_^2+^, Hg^2+^, Tl^3+^, Cd^2+^, Cr^3+^, Cr^6+^, Ag^+^, Cu^2+^, Ca^2+^, Mg^2+^, and Na^+^ [24]. Nevertheless, the vast majority of the developed assays are solution-based, utilizing complicated multi-step procedures and sophisticated instrumentation, which limits their utility for on-site, real-time applications.

There is, therefore, an urgent need for the development of analytical techniques and devices for the on-site and real-time monitoring of heavy metal ions in potable water, which will complement or completely replace conventional detection procedures. To this end, several examples of microfluidic-based devices have been reported [25]. A representative example is the work by M. Zhang et al. [20], who have fabricated a digital microfluidic system with an electrochemical sensor for on-site detection of heavy metal ions in tap water, or the work by Rotake et al. [26], who have developed a portable microfluidic platform using a microcantilever-based piezoresistive sensor for Cd^2+^ detection with a limit of detection (LOD) of 2.78 pM. In another effort, Santangelo et al. [27] combined an epitaxial graphene (EG) sensor with a 3D-printed microfluidic for heavy metal ion detection. They reported a quite low LOD of 95 nM for Pb^2+^, which they attributed to the high sensitivity of the sensing material. However, such approaches can be further improved by increasing the device integration degree and simplifying the device fabrication methods.

In this work, a highly integrated microfluidic-based device incorporating a passive mixing unit (for sample pretreatment) and a detection unit with an array of electrochemical biosensors for real-time, label-free detection of heavy metal ions is presented. In addition, the device incorporates all the necessary electrical connections for the operation of the device and all these are realized on a single flexible polyimide (Kapton) substrate. The biosensor used in the detection unit has been presented previously by our group [28] and has been shown to exhibit high selectivity against Pb^2+^ as well as sensitivity down to 10 nM (well below the WHO recommendations). The device fabrication process is kept simple, and it is based on dry photoresist optical lithography for the formation of the mixing and the detection unit. The detection unit is an array of six pairs of gold (Au) interdigitated electrodes (IDEs) on which deposition of PtNPs is followed by immobilization of the DNAzymes. The fabrication method proposed here significantly enhances the integration scale, since sample preparation takes place on the chip using the integrated microfluidic mixing unit before the sample is forwarded to the detection unit. To showcase the proof of concept operation of the fabricated prototype device, real-time, label-free Pb^2+^ detection is demonstrated on the chip.

## 2. Materials and Methods

### 2.1. Polymeric Films

A laminated photosensitive dry resin (ORDYL SY 300) purchased from Resistechno (Lainate (MI), Italy) is used for the fabrication of the microfluidic channel using negative tone lithography to pattern the microfluidic channels in a Karl Suss MJB3 mask aligner with a broadband mercury lamp i-line 365 nm (exposure time was 40 s), while the sealing of the channels is achieved by a 50 μm thick piezo-adhesive polyolefin (StarSeal), purchased from Starlab International GmbH (Hamburg, Germany). Commercially available 2 mm thick Polymethyl methacrylate (PMMA) is used for the development of a transparent microfluidic device used for the mixing evaluation. A polyimide Kapton substrate of 125 μm thickness was purchased from Goodfellow Cambridge Ltd. (Huntingdon, UK).

### 2.2. Reagents

All reagents were obtained from Merck (Merck KGaA, Darmstadt, Germany). All buffers were prepared with deionized water (18.2 MΩ·cm at 25 °C resistivity) from a Millipore MilliQ system. Oligonucleotides employed toward the detection of Pb^2+^ ions were purchased from Integrated DNA Technologies, BVBA (Leuven, Belgium). Their sequences were GTTCGCCATCTGAAGTAGCGCCGCCGTATAGTGACT (Enzyme strand) and AGTCACTATrAGGAAGATGGCGAAC (Substrate strand). The substrate strand was modified at 5′ with an amino C6 linker to allow its immobilization onto the silanized sensor surfaces. For the immobilization of the substrate strand onto the sensor surfaces and their blocking with ethanolamine, a 1 Μ Phosphate buffer pH = 8.5 was employed. Enzyme strand hybridization with the immobilized substrate strand was accomplished using a 50 mM MOPS and 25 mM NaCl pH 7.5 buffer. The same buffer was used to make up the Pb^2+^ solutions with which the sensors were interrogated. Finally, glutaraldehyde was applied to PBS 1× pH 7.4 buffer.

### 2.3. Microfluidic Device Design and Fabrication Process

#### 2.3.1. Chip Design

**Figure 1:**
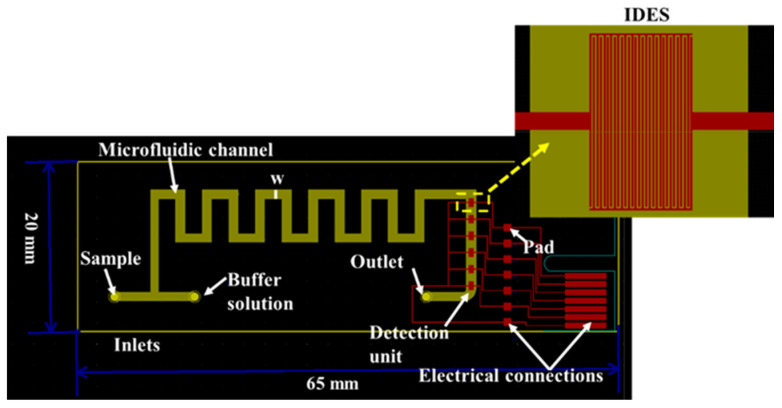
The microfluidic device design in KiCad software version 4.0.0-rc1. The mixing unit is a square wave channel, which is located before the detection unit. The detection unit is an array of electrodes with an inter-finger distance (electrode gap) of 10 μm, one next to the other at a distance of 1 mm. The electrical connections for the operation of the microfluidic device are also shown in red.

The microfluidic network is an important part of any portable, compact and lightweight on-site measurement device. For this purpose, the design and the fabrication of the chip were kept simple while maintaining a small footprint of 20 mm × 65 mm. The device consists of a microfluidic channel, 1 mm wide (w), with a mixing and detection unit (Figure 1). The former is necessary for the passive mixing of the sample (i.e., water) with a buffer solution prior to the detection of the heavy metal ions. Sample preconditioning is important to cancel out the effect of ionic composition and strength variations of real water samples. These differences in the concentration of monovalent ions like Na^+^, K^+^, and Cl^−^ and/or divalent ions such as Ba^2+^, Ca^2+^, Mg^2+^, and Fe^2+^ can affect the DNAzyme’s cleaving efficiency and hence sensor performance, leading to inaccurate measurements [29,30]. The mixing unit takes the form of a square wave channel with two inlets: one for the sample and the second for the buffer solution. This design has been found to offer an effective mixing index over a wide range of flow rates when compared to Τ shape, zigzag, multi-wave, loop, and mouth-shaped passive mixers [31]. In our case, the theoretical estimated mixing index is higher than 60% following the findings of the work of Chen et al. [31]. This could be further increased by adding more unit cells in the mixing unit. However, we wanted to keep the device footprint small. In Section 3.1, the mixing efficiency is evaluated by implementing a transparent device (for naked-eye estimation), while quantification is performed off-chip using absorbance measurements. The results clearly show that effective mixing is achieved (probably well above the theoretical estimated value).

The detection unit consists of an array of IDEs, which is placed after the mixing unit. Finally, the operation of the device is ensured either through a series of electrodes (red parallelograms), which are the same as those used for the Peripheral Component Interconnect Express (PCIe) located at the end of the device, or through seven electrode square contact pads located next to the detection unit, which can be used with spring-loaded pins.

#### 2.3.2. Fabrication Process

**Figure 2:**
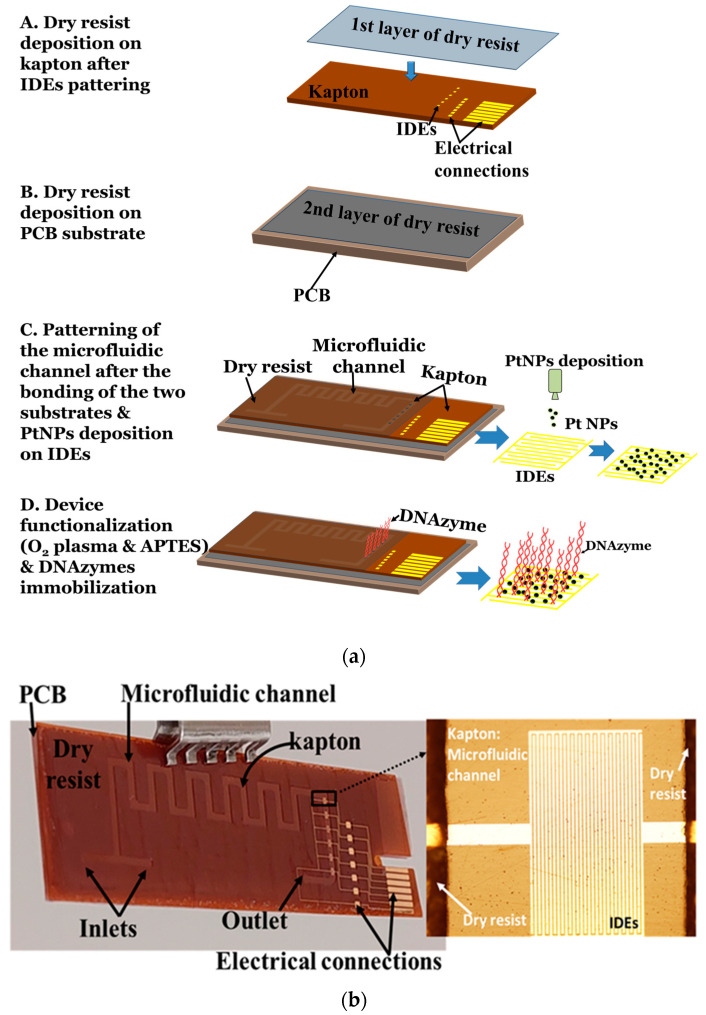
(**a**) Schematic representation of the device fabrication process, including the PtNPs/DNAzymes biosensor array fabrication procedure. The PtNP deposition procedure and the immobilization of DNAzymes in between the PtNPs are depicted. (**b**) Actual image of the final device. As an inset, we provide an optical microscopy image of the IDEs inside the detection unit of the device (10× magnification).

The device fabrication process was as follows. First, an array of six IDEs was fabricated on a 125 μm thick Kapton substrate using the e-gun technique, consisting of a 10 nm thick Titanium (Ti) layer (acting as an intermediate adhesive layer between substrate and gold) and a 40 nm thick Au layer. The IDEs were patterned on top of the substrate using photolithography, having an inter-finger spacing of 10 μm. Then, the 1st layer of a dry resist film, which is a laminated photosensitive film, is deposited on the Kapton substrate (Figure 2a, step A). The dry resist film is perfectly compatible with the substrate, while its thickness is 90 µm. To optimize the adhesion of the resist on the polyimide substrate, the Kapton substrate with the resist is passed through a laminator, the temperature of which is set at 105 °C.

Alongside, a 2nd layer of the dry resist film is deposited on a printed circuit board substrate (PCB) (Figure 2a, step B) again using a laminator. Finally, the two substrates (Kapton and PCB) are bonded. The PCB substrate improves the stability and the flatness of the flexible substrate while simultaneously enabling the handling of the device and the interconnection of the input and output of the solutions. Next, the microfluidic network is patterned on the 1st layer of the dry resist film. The device is immersed in the SY developer, which effectively removes the resist film from the region of the microfluidic channels, thus also exposing the Au IDEs (Figure 2a, step C). Finally, the device is baked at 110 °C for 30 min. The use of dry photosensitive films instead of liquid resins reduces the number of steps required for the microchannels patterning (i.e., spin coating, pre- and post-baking steps and any substrate etching required for pattern transfer) since the dry resist film has the thickness required to form the microchannel. Thus, using only one photolithography step, we can easily fabricate the microfluidic-based device. In addition, the exposure requirements during the photolithography step for the proposed design are minimal, and the step can be easily performed using a simple UV lamp.

After the microfluidic channel fabrication step, the PtNPs/DNAzyme biosensor fabrication procedure is as follows (Figure 2a, step C). The PtNPs with a mean diameter of 4 nm are deposited on top of the IDEs using a modified DC magnetron sputtering deposition system (Nanogen Magnetron DC Nanoparticle source manufactured by Mantis Deposition Ltd., Birmingham, UK). The sputtering process is a well-established technique for metallic nanoparticle synthesis, allowing the control of their mean diameter as well as their surface coverage [28]. The surface coverage of the PtNPs is close to 49%, which results in an interparticle distance of a few nm, whereas the resistance of the device is approximately hundreds of kΩ to a few MΩ.

Then, the surface of the device is functionalized (Figure 2a, step D). Prior to the chemical modification of the sensor surface, the device is treated with oxygen plasma at a custom-made ICP reactor using the following conditions: O_2_: 100 sccm, plasma power: 500 W, pressure: 10 mTorr, no bias voltage, duration: 2 min. Oxygen plasma treatment activated the surface of the polymer substrate by introducing hydroxyl groups (-OH) that are required for the subsequent silanization of the surfaces [32]. Similar plasma treatment protocols have been used to significantly improve biomolecule immobilization on other polymeric surfaces [33,34]. Using this plasma step, the homogeneity of the silanization step using 3-aminopropyl triethoxysilane APTES is enhanced. Surface silanization is accomplished by immersion in an aqueous APTES solution (5% *v*/*v*) for two hours, rinsing with copious amounts of water and thermal annealing at 110 °C for a further hour. Aqueous solutions of the silane are employed, contrary to the organic solvent normally used for silane deposition, due to limitations imposed by the polymer substrate. Further to this, the baking step serves in the annealing of the deposited silane molecules through the expulsion of water and the enhancement of their intra- and inter-crosslinking to the surface hydroxyls. The end result of surface silanization with APTES is the introduction of amine groups that are, in turn, additionally modified with glutaraldehyde. Glutaraldehyde treatment (5% *v*/*v* in PBS 1× pH = 7.4) is undertaken for one hour, rinsed with the same buffer and incubated with the amine-modified substrate strand (at a concentration of 5 μΜ for one hour in a humid chamber). The same buffer is employed to rinse off non-covalently attached surfaces, while any remaining amine groups on the silanized surfaces are blocked with a solution of ethanolamine (10 mM) for one hour. Hybridization of the enzyme strand of 10 μΜ is performed through its incubation with the substrate strand-modified surfaces for 1 h in a humid chamber.

The device is finally ready to be used after sealing with a polyolefin film coated on one side with a silicone adhesive. The schematic representation of the device fabrication process steps is shown in Figure 2a, while in Figure 2b, the final device with all the important parts (i.e., microfluidic channel and IDE array) is depicted.

### 2.4. Mixing Efficiency Evaluation

For the evaluation of the mixing unit, a transparent device with the same design was placed inside a chip holder to enable the solutions to flow using silicone tubes and a syringe pump. Then, absorbance measurements of yellow and blue food dyes (Vahine, a brand of McCormick & Company, Inc., Hunt Valley, MD, USA) were performed using Lambda 35 UV/Vis Spectrophotometer (Perkin-Elmer, Waltham, MA, USA). The absorbance spectrums of the initial solutions, as well as of the solution after mixing, were recorded and compared with the absorbance spectrums of food colorings existing in the literature [35].

### 2.5. Experimental Setup for the Electrical Detection of Pb^2+^

**Figure 3:**
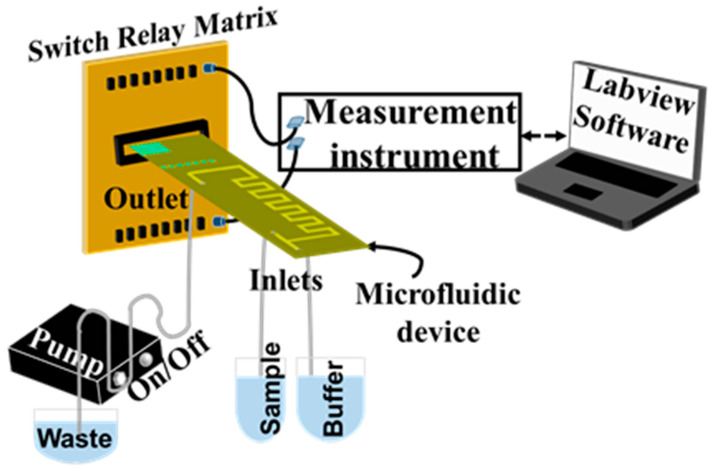
Schematic representation of the overall experimental setup used for the detection of heavy metal ions, which can be easily integrated into a complete portable water analysis system. A peristaltic pump is used for control of flow, whereas a switch relay matrix enables the simultaneous measurement of multiple sensors.

For the electrical detection of Pb^2+^, the device is placed in a chip holder to enable fluidic connections and a peristaltic pump (INSTECH P.625/900,143) is used to circulate the liquids inside the microfluidic channel, while the response of the device is obtained via a switch relay matrix connected to an HP34401 Digital Multimeter and recorded by the Labview program (Figure 3).

## 3. Results and Discussion

### 3.1. Evaluation of the Passive Mixing Unit Using a Transparent Device

**Figure 4:**
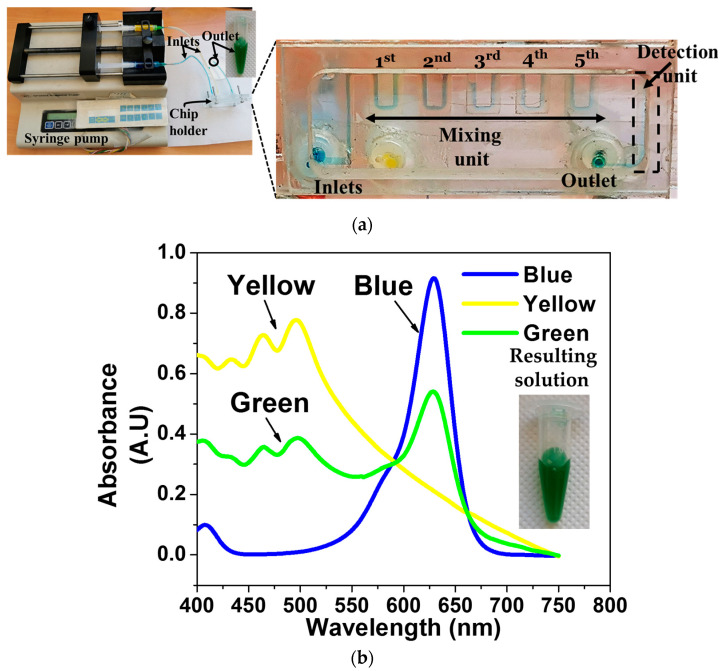
(**a**) Evaluation of the passive mixing unit using the transparent PMMA device. (**b**) The absorbance spectrum of the starting blue and yellow solutions and the final (green) solution after mixing. The absorbance spectrums are in agreement with typical absorbance spectrums reported in the literature for the food coloring solutions like the ones used here, verifying the successful mixing of the two solutions.

First, the device’s ability to mix two different solutions through the microfluidic-based mixing unit was evaluated. For this purpose, the same microfluidic channel was fabricated on a 2 mm thick, transparent PMMA substrate, which allows real-time observation of mixing as the initial solutions flow into the mixing channel. Yellow and blue food coloring solutions were used as reference solutions. These two solutions are inserted from the two inlets, and they are mixed before the detection unit. In particular, Figure 4a shows that the two initial solutions (yellow and blue) start to mix after the third square unit and the solution color tone changes, indicating effective mixing. The final solution coming out of the channel outlet was green, verifying the successful mixing of the two solutions (Figure 4a). To further strengthen and quantify this naked-eye observation, we measured the absorbance spectrum of the mixed green solution as well as of the two solutions (yellow and blue) used for the mixing evaluation (Figure 4b). It was found that the main peak for the yellow solution is observed between 450 and 500 nm (as expected); for the blue-colored solution, the main peak is observed at 600–650 nm (again as expected), whereas the mixed green solution exhibited, as expected, two main peaks at approximately 450–500 nm and at 600–650 nm, similarly to what is reported in the literature for green food colorings (Figure 4b) [35].

### 3.2. Sensing Principle and Real-Time Measurement of Pb^2+^ Using the DNAzyme Biosensor

The sensors’ principle of operation relies on the differences in charge transport observed across single- and double-stranded DNA and the coverage of the surfaces with nanoparticles. By tuning the deposited nanoparticles’ size and inter-particle distance, an intermediate conductivity state can be attained, whereby electron hopping in between adjacent nanoparticles occurs and is further enhanced through the bridging effect of linkers, including that of organic molecules [36]. When the coverage of the sensor surfaces with nanoparticles exceeds this percolation threshold, electrical conductivity jumps up several orders of magnitude due to the formation of continuous electron paths or conducting networks that are insensitive to the presence of linkers. In the opposite case, placing the nanoparticles too far apart results in an insulating composite that does not allow the transport of charge.

In the case of the deposited PtNPs’ 2D network, the conductivity of the sensors is affected by the ability of the immobilized DNAzymes to transfer electrons across. Their conductivity, and by consequence, that of DNA and nucleic acids in general, is presumably the outcome of extensive π-stacking between base pairs and varies depending on the base composition and, hence, the flexibility of the molecule as well as the temperature. Despite the ambiguous or even conflicting data generated on DNA conductivity, depending on the technique employed to study charge transport phenomena, a number of different mechanisms have been proposed, with electron hopping and tunneling being the most prominent ones [36,37,38,39,40]. Nevertheless, and irrespectively of the causative reason underlying DNA conductivity, single- and double-stranded species display markedly different electrical behaviors, with the former acting as insulators, whereas double-stranded DNA behaves as a molecular wire, facilitating charge transport. DNAzymes, like the GR5 DNAzyme employed in this work, are double-stranded chimeric nucleic acids that, upon presentation with a heavy metal ion, exhibit a self-catalytic cleavage of one of the two strands (substrate). Therefore, it is anticipated that the electrical conductivity of the cleaved DNAzyme will be significantly decreased relative to that of the uncleaved DNA duplex.

Herein, the aforementioned phenomena were exploited to construct a sensor capable of translating Pb^2+^ detection and the resultant DNAzyme cleavage into a measurable change in the resistance of the device. To achieve this, and based on the results previously obtained on the optimal nanoparticle surface coverage, the IDE arrays were modified with a network of nanoparticles covering 49% of the surface, while their mean diameter was 4 nm (SD ± 1.5 nm) [28]. The obtained PtNP surface coverage is right below the percolation threshold of the system and results in an inter-particle distance of a few nm with resistance ranging from 500 kΩ to 2 MΩ.

The gap between the nanoparticles is linked by the immobilized DNAzymes, whose end-to-end distance can be estimated based on models describing semiflexible polymers. Based on the most recent simulation results, both the end-to-end distance of the GR5 DNAzyme and its contour length are roughly equal to 6 nm [41], a value approximating the inter-particle distance of the PtNP network deposited on top of the IDE arrays. It is plausible, therefore, to assume that the double-stranded DNAzyme, which is immobilized onto the surfaces through the substrate strand, facilitates charge transport between the nanoparticles. Electron transport is disrupted once the duplex is cleaved, leaving only one segment of the substrate strand still immobilized on the PtNPs’ surface. Practically, this leads to a twofold increase in the device’s resistance, as the remaining segment is not only physically incapable of ‘wiring’ the gap between the nanoparticles due to its decreased length, but also due to the fact that it is single-stranded.

**Figure 5:**
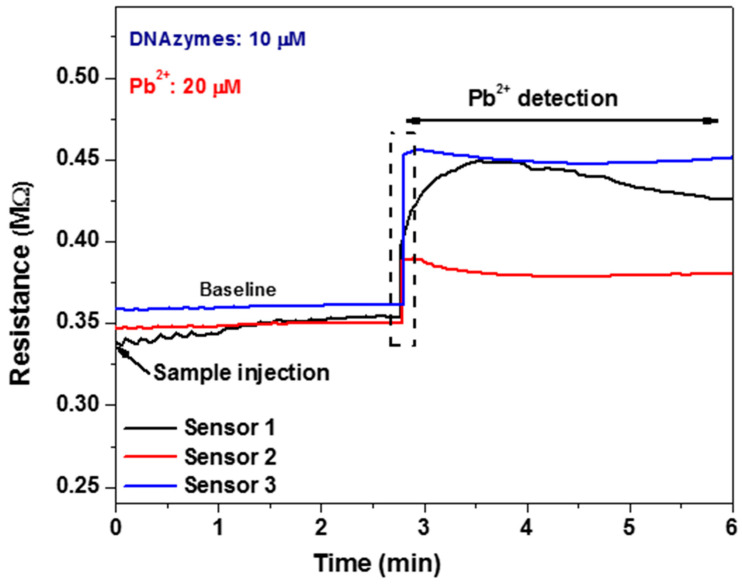
Real-time measurement of Pb^2+^ using the proposed DNAzyme biosensor array (Sensor 1 response: black curve, Sensor 2 response: red curve, and Sensor 3 response: blue curve). In the beginning, while the buffer solution is flowing over the detection unit, the resistance reaches a steady state, and the baseline curve is obtained. Then, the sample is injected (t = 0 min) and reaches the detection unit in 2 min, causing a resistance increase due to the cleavage of the DNAzyme double strand caused by the presence of Pb^2+^ (reaction takes place within 1 min). This increase is recorded by all the three sensors forming the detection unit.

For the electrical detection of Pb^2+^, the following protocol is followed. First, the buffer solution is injected inside the channel, using one of the two inlets, until the resistance value of the sensors reaches a steady state, which is the baseline for the measurement. Subsequently, the sample containing Pb^2+^ (Pb(NO_3_)^2^) is injected and reaches the detection unit in 2 min after being passively mixed with the buffer solution. The real-time measurement of Pb^2+^ using the proposed array of the three DNAzyme biosensors is provided in Figure 5 (Sensor 1 response: black curve, Sensor 2 response: red curve, and Sensor 3 response: blue curve). As expected, the presence of a 20 μM Pb^2+^ leads to the cleavage of the DNAzyme double strand, and the resistance recorded by all sensors increases within 1 min, with the entire detection protocol lasting 6 min. The resistance increase is attributed to the change in the conductive path formed by DNA enzymes and the nanoparticles. Although 20 μM is higher than the WHO standards (10 μg L^−1^) [42], it demonstrates the proof of concept of the device. Furthermore, as discussed in a previous publication by our group [28], the proposed biosensor platform has been calibrated against varying concentrations of Pb^2+^, showing linear response between 10 nM and 1 μM and small to no cross-reactivity when tested in varying concentrations of non-specific divalent metal ions such as Mg^2+^ and Cu^2+^. It is, therefore, safe to claim that the proposed sensing scheme is responsive to lead ions only.

Our vision is to use the proposed device for the simultaneous detection of different heavy metals, such as Pb^2+^, Cd^2+^, Hg^2+^, and Cr^6+^. This can be achieved by the fabrication of a sensor array consisting of different biosensors, each one being selective to a specific metal ion. The design of the proposed device enables such multiplexing, since it consists of an array of six sensors that can be suitably modified to detect different heavy metal ions. In addition, it is also intended to integrate the few peripherals (the peristaltic pump, the electrical connections, and the PC or tablet for control) into a completely portable platform for the on-site detection of heavy metal ions (Figure 3). Our approach with the integration of the mixing unit for sample preparation as well as the biosensors into the microfluidic device, together with the corresponding accompanying electronic readout system, can enable the realization of a fully portable chemical analysis system capable of direct environmental monitoring in the area of interest and the rapid (on the order of a few minutes) detection of hazardous environmental pollutants. The proposed device is based on an array of DNAzymes and nanoparticle biosensors, which allows the multiplexed detection of more than one heavy metal ion through the functionalization of the sensors with different DNAzymes. At the same time, the acquisition of multiple measurements for a single sample and the estimation of the concentration of the heavy metal ion in an unknown sample using the averaged sensor response permits any deviations in sensor response. In principle, the device can exhibit a response equivalent to that of established water analysis techniques since, in our previous work [28], we have shown a successful detection and linear response for concentrations ranging between 10 nM and 1 μM. We are already working in this direction, trying to promote this “advanced monitoring system” that can fit in a small suitcase for point-of-interest water analysis in wastewater treatment plants, energy industries, and food industries.

## 4. Conclusions

The development of a microfluidic device using a flexible polyimide substrate (Kapton) incorporating the two basic components required for its operation, i.e., a passive mixing unit and PtNPs–DNAzyme-modified IDE biosensors for on-chip heavy metal ion detection, is presented. The proposed device is based on microfluidic technology using a combination of electrochemical sensing with biorecognition elements (DNAzymes). The device fabrication procedure is kept simple and cost-effective using ready-to-use materials (i.e., dry resist films) and well-established microelectronic processes commonly used in the industry (i.e., photolithography and sputtering). The successful proof-of-concept detection of Pb^2+^ ions is demonstrated, indicating the ability to detect heavy metal ions in real time and in 6 min. The proposed device can operate with few peripherals since the electrical connections are embedded in the device, enabling easy connection and control, and water sample preparation and detection have been designed to be accomplished passively using only one peristaltic pump with a small footprint. In addition, the device consists of an array of biosensors, which can be used in order to detect different heavy metal ions, enabling multiplexing. It is, therefore, evident that the results of this work can pave the road towards a portable platform enabling label-free real-time measurements of water safety at the point of interest.

## Data Availability

All data created are included inside the manuscript.
